# Incidental Adrenal Nodules and Masses: The Imaging Approach

**DOI:** 10.1155/2015/410185

**Published:** 2015-04-29

**Authors:** J. Willatt, S. Chong, J. A. Ruma, J. Kuriakose

**Affiliations:** ^1^University of Michigan Health System, Ann Arbor, MI 48109, USA; ^2^Veterans Administration Hospital, Ann Arbor, MI 48105, USA

## Abstract

Adrenal nodules are detected with increasing frequency. The National Institute of Health (NIH), American College of Radiology (ACR), and the American Association of Clinical Endocrinologists and American Association of Endocrine Surgeons (AACE/AAES) have produced guidelines for the management of incidental adrenal nodules. This review provides a summary of the consensus radiologic approach to these nodules.

## 1. Introduction

The burgeoning use of cross-sectional imaging in daily medical practice has led to a proportional increase in the burden of incidentally discovered nodules in the adrenal glands [1]. Adrenal nodules are seen in approximately 5% of abdominal CT scans [1, 2]. Endocrinologists and radiologists have developed strategies for managing these “incidentalomas” [3–5].

Three questions need to be answered when defining a diagnostic pathway for incidentally discovered adrenal nodules [5].

1. Is the lesion malignant, and if so, is it a primary adrenocortical cancer (ACC), or is it a metastasis?
2. Is the lesion a pheochromocytoma?
3. If the lesion is an adrenal adenoma, is it functioning?

The National Institute of Health (NIH) [6], the American College of Radiology (ACR) [7], and the American Association of Clinical Endocrinologists and American Association of Endocrine Surgeons (AACE/AAES) [8] have each produced guidelines from which a broad consensus can be reached. This paper focuses on the radiological component of the diagnostic work-up.

## 2. Is the “Incidentaloma” an Adrenocortical Carcinoma or Metastatic Disease?

In patients with a known malignancy, the likelihood of an adrenal nodule being malignant is approximately 25–36%. However, in the population without a known malignancy, the prevalence is less than 0.5% [9–12]. Therefore a strategy for characterizing these nodules at minimum cost and without invasive tissue sampling is important. Techniques have been developed primarily using Computed Tomography (CT) and Magnetic Resonance Imaging (MRI) which spare the patient the need to undergo further testing for possible malignancy.

However, the assessment of size remains the single most useful determinant of the nature of silent adrenal lesions [9, 13] as survival rates following adrenalectomy are greater for smaller masses than for larger ones [14]. Although the specificity is low (42%), a 4 cm cutoff as an indication for surgery is sensitive (93%) [14], and this is therefore used as a threshold criterion alongside other factors such as imaging features, hormonal data, patient age, and the presence of abdominal pain.

## 3. Diagnostic Approach

The etiology of adrenal nodules can be determined on both CT and MRI for several entities without further work-up.

1. An adrenal myelolipoma is a benign tumor containing mature adipose tissue and hematopoietic tissue. They are usually asymptomatic. On rare occasions, they can become symptomatic as they can grow large. They can also spontaneously bleed. They can be diagnosed with very high specificity by CT and MRI as these are the most common adrenal lesions to contain large amounts of macroscopic fat. On MRI the fat components of most myelolipomas will demonstrate fat suppression [15]. On CT, if there are regions measuring less than −20 Hounsfield units (HU) signifying fat then the diagnosis can be made [16].
2. Adrenal hemorrhage frequently occurs without symptoms. A history of trauma or anticoagulation is helpful. On CT they can appear as round or oval masses, often with adjacent inflammatory change. They decrease in size over time, but a residual organized hematoma can persist which may contain calcifications [17]. They demonstrate HU of 50–90 on noncontrast CT. On MRI they can be of variable signal intensities on T1-w and T2-w imaging, depending on the chronicity. They do not enhance following intravenous gadolinium chelate or iodinated contrast administration. CT and MRI (particularly with subtraction imaging) use enhancement characteristics to distinguish a simple adrenal hemorrhage from an adrenal mass [18].
3. Adrenal cysts are not common. They show similar characteristics to renal or liver cysts. HU can be between −10 and 20, and they are hypointense on T1-w imaging and hyperintense on T2-w imaging. They do not enhance after contrast administration.
4. Adrenal adenomas represent 75% of the adrenal incidentalomas found on CT. Adenomas are benign tumors containing a variable amount of intracytoplasmic lipid. This enables the use of HU measurements on CT, or the chemical shift artifact on MRI, to characterize adenomas without the need for intravenous contrast [19–21]. If the lesion demonstrates HU of less than 10 on unenhanced CT then it can be declared a lipid rich adenoma with 98% specificity [22].

MRI can also be used to diagnose lipid rich adrenal adenomas using the dual gradient echo sequence. If an adrenal nodule loses signal on opposed imaging in comparison with in-phase imaging then it can be declared an adenoma [23]. More often than not this is seen with the naked eye. However, for more equivocal cases a threshold of 16.5% signal loss can be used [24]. This is known as the signal intensity index. Chemical shift MRI (CS-MRI) is more sensitive than unenhanced CT for intracytoplasmic lipid content and can diagnose many of the nodules which demonstrate HU of between 10 and 30 as lipid rich adenomas [25].

25% of adrenal adenomas contain insufficient intracytoplasmic lipid to conform to the noncontrast features described for lipid rich adenomas. These require further characterization with contrast-enhanced CT [26–29]. The “adrenal protocol” CT begins with an unenhanced set of images. If the lesion does not demonstrate HU of 10 or less then intravenous iodinated contrast is given. The technique is based on the enhancement characteristics of adenomas which lose contrast more rapidly than metastases. A contrast “washout” calculation is therefore performed which uses the HU of the nodule before contrast injection, 60–70 seconds after contrast, and at a “delayed” phase of 15 minutes. The “absolute percentage of washout” (APW) is calculated using the following formula:

(1) APW=enhanced  HU−15-minute  delayed  HU÷enhanced  HU−unenhanced  HU×100.

If the absolute washout is greater than 60% then this classifies the lesion as a benign adenoma [28].

If a nodule is noted on a single phase contrast-enhanced CT, then a relative percentage washout (RPW) can be calculated if a 15-minute delayed phase is subsequently performed:

(2) RPW=enhanced  HU−15-minute  delayed  HU÷enhanced  HU×100.

If the relative washout is greater than 50% then this classifies the lesion as a benign adenoma [29].

These formulae have been validated demonstrating accuracy of more than 96% [30].

Caution should be applied when patients have histories of renal cell carcinoma or hepatocellular carcinoma, as metastases from these primary tumors can, on rare occasions, be mistaken for adrenal adenomas on CS-MRI or on adrenal washout CT [31–33]. Interval growth differentiates these malignant entities from adenomas. Other lesions that have been reported to demonstrate intracytoplasmic or even gross lipid are pheochromocytomas, adrenal lymphangiomas, and adrenocortical carcinomas [34–39]. Again lesion growth, size, and the presence of calcification combined with clinical suspicion and laboratory values are helpful in distinguishing these tumors from adenomas.

## 4. Pheochromocytoma and Malignancy

If a nodule cannot be diagnosed to be one of the benign entities above, a history of current or previous malignancy should be sought. Pheochromocytoma, primary adrenocortical carcinoma, and metastasis should now be excluded.

10% of pheochromocytomas are bilateral and 10% are malignant. On CT, they can be of homogeneous or heterogenous density [40, 41]. On MRI they are often, but not always, of high signal intensity on T2-w imaging [40] and enhance rapidly. As they have been shown to demonstrate similar washout characteristics to adenomas [42] further evaluation with urine or plasma metanephrine levels is necessary, and positron emission tomography (PET) can be useful with either FDG (18-fluoro-2-deoxy-D-glucose) or MTO (11C-metomidate) [43].

Adrenocortical carcinoma is rare, with a prevalence of 12 in 1 million, and has a poor prognosis [44]. Metastases are more common than ACC [45]. When a large adrenal mass is found incidentally then a search for a primary tumor or for further metastatic disease can be performed, potentially including the use of PET/CT [46].

ACC comprises less than 2% of adrenal incidentalomas measuring less than 4 cm and 25% of lesions measuring greater than 6 cm [6]. A cutoff of 4 cm can be used to decide if an adrenal mass is benign or malignant with greater than 90% sensitivity [13, 47, 48]. Other findings suggestive of malignancy are an irregular border, central areas of necrosis, and invasion of adjacent structures including the IVC and the liver [49]. However, metastatic disease is rare in patients without a known history of malignancy [11, 50]. Lymphoma, melanoma, lung, breast, renal, and gastric tumors are the most common origins, and, in patients with these diagnoses, an adrenal mass is likely to be metastatic [51].

**Figure 1:**
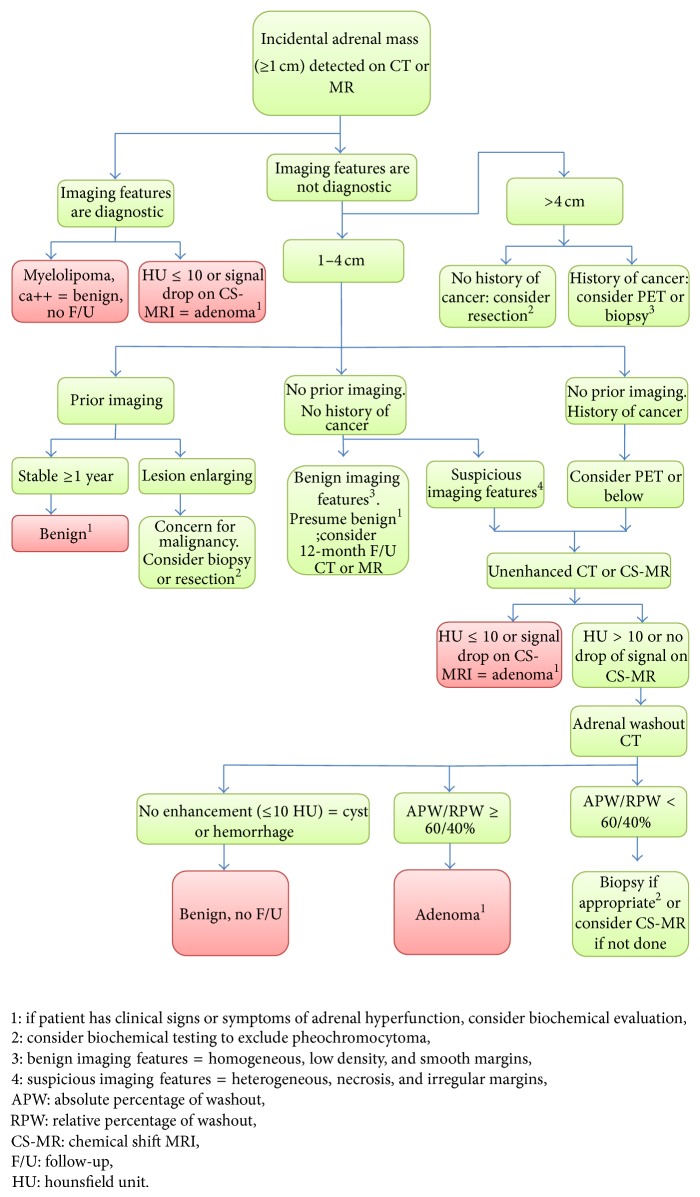
ACR guidelines flow chart [52].

In 2010 the ACR published a white paper which included a flow chart on incidental adrenal nodule findings [52]. The flow chart (Figure 1) can be summarized as follows.

1. If a nodule of any size can be diagnosed as a myelolipoma or a lipid rich adenoma then no further imaging is warranted.
2. A nodule measuring 4 cm or less which demonstrates benign imaging features such as low density, homogeneity, and smooth margins can be followed with a single further CT or MRI if there is no prior imaging with which to compare the size. If it is stable in size after one year it can be regarded as benign and does not need any further follow-up.
3. If a 1–4 cm nodule demonstrates suspicious imaging features such as heterogeneity, necrosis, or irregular margins, or if it can be shown to be growing either retrospectively or at follow-up, then an adrenal nodule work-up should commence. This would include noncontrast CT or MRI, if not performed already. If the nodule is not diagnosed on these studies as a lipid rich adenoma, then an adrenal washout CT can be performed to see if it is a lipid poor adenoma. If it enhances and is therefore not a cyst but does not meet the washout criteria for an adenoma, then the level of suspicion should be raised and biopsy considered.
4. If an adrenal mass measures more than 4 cm then a history of malignancy should be sought. If there is no history, then resection should be considered given the incidence of ACC in this group. Care should be taken to biochemically exclude a pheochromocytoma before intervention. If there is a history of malignancy, then PET/CT can be used to establish the location(s) of further disease, and, in the absence of a tissue diagnosis, biopsy can be considered.

## 5. Adrenal Biopsy

Biopsy of the adrenal glands can be technically challenging because of their locations, between the right kidney and liver or the left kidney and spleen or stomach. An axial approach will often transgress the diaphragm. The lower ribs extend below the level of the adrenal glands making visualization with ultrasound difficult. There is very little literature on adrenal biopsy in comparison with that of the liver or kidneys. Fine needle aspiration (FNA) has often been preferred over core biopsy because of the risks involved.

Adrenal biopsy is only performed, therefore, when the diagnostic imaging pathways have failed to lead to a diagnosis, and even then masses over 4 cm in size are often resected without prior tissue diagnosis. Pneumothorax, severe hemorrhage, and hypertensive crisis have been reported following adrenal nodule FNA [53, 54]. Biopsy is generally reserved for patients with a history of cancer to determine if the mass is metastatic from the known primary tumor.

## 6. Pitfalls

Collision tumors, where adjacent but histologically distinct entities are found within the same adrenal nodule, are rare, but recognized [55, 56]. These often consist of a contiguous adrenal adenoma and a metastasis from a remote primary malignancy [55], but other combinations including adenoma with myelolipoma, hemangioma with adenoma, and adrenocortical carcinoma with myelolipoma are seen. Vigilance is required on the part of radiologists to ensure that all components of a nodule are assessed.

Before biopsy, ablation, or surgery, pheochromocytoma should be ruled out biochemically [6]. Even after ruling out pheochromocytoma, hypertensive crises are common when adrenal procedures are performed. Monitoring with an arterial line and close availability of antihypertensives are required.

## 7. Biochemical Evaluation

Patients who have an adrenal incidentaloma need to undergo a clinical examination to exclude a functioning tumor [6, 8]. If the lesion is not a myelolipoma then biochemical evaluations for autonomous cortisol production, for pheochromocytoma, and, in patients who are hypertensive, for primary hyperaldosteronism should be performed at detection [4] and annually for 5 years.

## 8. Imaging Follow-Up

Approximately 15% of adrenal incidentalomas increase in size during follow-up. Known malignant masses can remain unchanged in size over extended periods. In one study 12% of malignancies did not increase in size over 36 months [57]. Follow-up of adrenal nodules with CT is therefore controversial [4, 50]. However, the AACE/AAES guidelines suggest that for a nodule measuring more than 1 cm and less than 4 cm, repeat imaging with noncontrast CT should be performed at 3–6 months and annually for one to two years [8]. If the mass grows or becomes hormonally active then adrenalectomy should be performed.

Patients with masses measuring more than 4 cm should undergo evaluation for adrenalectomy.

## 9. Conclusion

Incidentally discovered adrenal nodules measuring 10 mm or less in patients without a history of cancer are followed up clinically, but not radiologically, unless a biochemical abnormality is discovered. Nodules measuring between 10 mm and 4 cm should undergo radiological evaluation until they are diagnosed. If they cannot be diagnosed then they can be followed with imaging for up to two years. Masses measuring greater than 4 cm, unless they are determined to be benign and they are asymptomatic, are removed surgically. Biochemical evaluation for pheochromocytoma should be carried out before biopsy or surgery is performed.
